# Supplementary material for: Analysis and differentiation of tobacco-derived and synthetic nicotine products: Addressing an urgent regulatory issue
Source: PLoS One. 2022 Apr 14;17(4):e0267049. doi: 10.1371/journal.pone.0267049 (PMC9009602; doi:10.1371/journal.pone.0267049)
Supplement: S1 Table — (PDF) [file pone.0267049.s001.pdf]

S1 Table. Tabulated analyte data for tobacco-derived and synthetic nicotine samples. Where analytes were determined in triplicate, the values are presented as "average ± standard deviation".

				Tobacco-Derived Nicotines (TDN)				Synthetic Nicotines (SyN)			
**Analyte Group**	**Analyte (unit of measure)**	**LOD**	**LOQ**	**TDN-1**	**TDN-2**	**TDN-3**	**TDN-4**	**SyN-1**	**SyN-2**	**SyN-3**	**SyN-4**
Nicotine Degradants	Anabasine (µg/g)	0.100	1.00	39.3 ± 1.2	ND	ND	768 ± 17	ND	ND	ND	ND
	Anatabine (µg/g)	0.050	0.50	14.3 ± 0.1	ND	0.0823[a] ± 0.0126	910 ± 17	ND	ND	ND	0.0690[a] ± 0.003
	beta-Nicotyrine (µg/g)	0.027	2.65	ND	ND	ND	81.1 ± 2.4	ND	ND	20.4 ± 1.6	ND
	Cotinine(µg/g)	0.100	1.00	884 ± 5	161 ± 2	378 ± 2	2313 ± 36	150 ± 2	141 ± 2	2055 ± 30	378 ± 14
	Mysomine (µg/g)	0.099	0.99	1340 ± 13	243 ± 7	366 ± 4	2806 ± 47	290 ± 3	285 ± 3	2171 ± 22	376 ± 18
	Nicotine *N*-Oxide (µg/g)	0.010	0.10	109 ± 1	5.89 ± 0.58	17.5 ± 0.7	447 ± 8	8.21 ± 0.16	2.65 ± 0.13	85.1 ± 2.5	11.8 ± 3.8
	Nornicotine (µg/g)	0.099	0.99	399 ± 8	12.8 ± 0.4	7.61 ± 0.51	588 ± 6	19.2 ± 0.7	16.0 ± 0.5	46.3 ± 1.5	13.1 ± 0.6
Metals	As (ng/g)	9.91	49.6	ND	ND	ND	ND	ND	ND	ND	ND
	Be (ng/g)	2.18	19.8	ND	ND	ND	ND	ND	ND	ND	ND
	Cd (ng/g)	0.991	4.96	ND	ND	ND	ND	ND	ND	ND	ND
	Cr (ng/g)	9.91	49.6	ND	ND	ND	ND	ND	10.3[a] ± 0.7	ND	ND
	Co (ng/g)	0.496	4.96	ND	ND	ND	ND	ND	ND	ND	ND
	Cu (ng/g)	24.8	74.3	ND	ND	ND	205 ± 8	ND	ND	ND	ND
	Fe (ng/g)	99.1	198	ND	ND	ND	115 ± 39	ND	ND	ND	ND
	Pb (ng/g)	2.48	9.91	ND	ND	ND	ND	ND	ND	ND	ND
	Ni (ng/g)	4.96	9.91	ND	ND	ND	ND	ND	ND	ND	ND
	Se (ng/g)	3.84	19.7	ND	ND	ND	ND	ND	ND	8.38[a] ± 1.9	ND
	Ag (ng/g)	0.991	4.96	ND	ND	ND	ND	ND	ND	ND	ND
	Sn (ng/g)	9.91	49.6	ND	ND	ND	ND	ND	ND	9.72[a] ± 3.65	ND
	Zn (ng/g)	49.6	99.1	ND	ND	1181 ± 7	92.0[a] ± 8.8	56.7[a] ± 5.6	ND	ND	ND
	Pd (ng/g)	90.0	200	ND	ND	ND	ND	ND	ND	ND	ND
Chiral Nicotine	(*S*)-Nicotine (%)	NA	NA	99.4	99.3	99.4	99.2	50.2	99.1	99.7	99.1
	(*R*)-Nicotine (%)	NA	NA	0.6	0.7	0.6	0.8	49.8	0.9	0.3	0.9
Chiral Nornicotine	(*S*)-Nornicotine (%)	NA	NA	67.2	85.9[b]	80.9[b]	68.7	50.4	100.0	100[b]	100[b]
	(*R*)-Nornicotine (%)	NA	NA	32.8	14.1[b]	19.1[b]	31.3	49.6	ND	ND[b]	ND[b]
Radiocarbon (^{14}C)	Percent Modern Carbon (%)	NA	NA	101.5	101.5	100.8	104.7	35.3	35.9	34.7	35.9

Notes

A superscipt "a" denotes a value below the limit of quantitation (LOQ)

A superscript "b" indicates the sample extract had very poor chromatography that prevented accurate quantitation

ND indicates no analyte was detected, i.e. was below the limit of detection (LOD)
