# Supplementary material for: Analysis and differentiation of tobacco-derived and synthetic nicotine products: Addressing an urgent regulatory issue
Source: PLoS One. 2022 Apr 14;17(4):e0267049. doi: 10.1371/journal.pone.0267049 (PMC9009602; doi:10.1371/journal.pone.0267049)
Supplement: Attachment — Submitted filename: Response to Reviewers.pdf [file pone.0267049.s004.pdf]

Response to Reviewers

We are grateful to the Academic Editor and Reviewers who took the time to assess our manuscript and provide helpful feedback. Below are our point-by-point responses to the comments and suggestions that were made.

Responses to the Academic Editor Comments

Comment 1

Minor revisions were made to the manuscript formatting to be in compliance with the journal guidelines. One major revision was the replacement of Table 1 with a figure (Fig 1) due to the presence of graphical objects. The numbering of subsequent figures was adjusted accordingly.

Comment 2

The authors thank the Academic Editor for their timely response to our request for clarification on this comment. As suggested, the financial disclosure statement has been updated in the cover letter to be:

"No specific funding was provided for this work. No part of this work has been funded by a tobacco company or similar entity."

Comment 3

No response required, see Comment 6.

Comment 4

All raw data for the analyses that were performed in triplicate (nicotine degradants and metals) have been included in the Supplementary Materials as an Excel workbook. Raw data for analyses performed in singlicate (chiral and radiocarbon analysis) were already included in S1 Table. The following statement has been added to the cover letter:

"All associated raw data can be found either in S1 Table or in the Excel workbook (Raw Data for Nicotine Degradants and Metals Analyses.xlsx)."

Comment 5

The manuscript submission data has been edited to include Jacob Hilldrup; we apologize for the inadvertent omission.

Comment 6

The authors thank the Academic Editor for their timely response to our request for clarification on this comment. As suggested, the Competing Interested statement has been updated in our cover letter to include the additional text indicated in red below:

"The authors are paid employees of their respective companies and do not claim any competing interests. Enthalpy Analytical, LLC, a wholly owned…"

Comment 7

The reference list has been reviewed and verified as requested. One change was the addition of a reference to a Politico article on synthetic nicotine that was released during the initial review period.

Responses to Reviewer 1 Comments

We thank the reviewer for their comments and appreciate that they feel the study will be of use to the scientific and regulatory community. Our responses to the specific points are given below:

Specific point 1

"*However, at least in the view of this reviewer, there remains a constraint that the authors do not make clear to the reader: The residual number for %-bio-carbon (e.g., in Figure 5) for the SyN-# samples likely depends on the method of synthesis.*"

The reviewer is correct that the %-biocarbon values for SyN samples may be dependent upon their route of synthesis and the reagents used, and as such may differ significantly from those we assessed. We have amended the text to clarify that different %-biocarbon values may be possible depending on the synthetic route and reagents used. Specifically:

Line 349:

"…of the two. However, it should be noted that this is predicated on the SyN used returning radiocarbon results that fall within the pMC value ranges we have observed during this study. The radiocarbon content of SyN could potentially be affected by the synthetic pathway and origin of the chemical ingredients used. Consequently, there may be SyN on the market, either currently or in the future, that does not possess a similar ^{14}C content to those analyzed here. As such, it is recommended that the assessment of the relative amounts of TDN and SyN in a mixture of the two be for qualitative purposes only."

We do note, however, that the samples we looked at represent the major suppliers of synthetic nicotine to the US market. Furthermore, since this work was performed, Enthalpy has tested additional samples for radiocarbon analysis and we have not seen any product with a %-biocarbon result that differs from those in this study, either being below 40% or at 100%.

Specific point 2

"*Thus, the application of a calibration curve (e.g., Figure 6) may lead to an incorrect number for %-tobacco derived nicotine.*"

The reviewer is correct in this assertion; however, it was not our intent that calibration curves would be generated and used to determine the SyN-to-TDN ratio of a mixture. Our intention for this experiment was merely to demonstrate that such a blended mixture would return a radiocarbon result that would be the proportion-weighted sum of their individual values. As such, a radiocarbon result somewhere between the two extremes we've observed would be suggestive of the test sample being a mixture of SyN and TDN, rather than one or the other. Taking into account the reviewer's previous point, a SyN with a significantly different (and higher) biocarbon value could give a misleading identification as a mixture. Given that we have yet to observe such a SyN, however, the burden would be upon the manufacturer to prove they are using a genuine synthetic nicotine. The following text was added to clarify this:

Line 361:

"As such, it is recommended that the assessment of the relative amounts of TDN and SyN in a mixture of the two be for qualitative purposes only."

Specific point 3

"*Thus, their method is not as definitive as suggested, and this limitation should be made clear, both in the Discussion and the Abstract. Alas, the goal of a final and definitive method for characterizing all these samples remains elusive.*"

If taken from the perspective of a method that could quantify the amount of TDN and SyN in a given sample, then the reviewer is correct. However, from the current regulatory standpoint it only needs to be shown that the sample in question does not contain any TDN to avoid regulatory action. The method as described allows for this since if the result is consistent with those of known SyN samples then it is confirmed to be synthetic in origin and no action can be taken. If any result above 40% is obtained, then it must be proven by the manufacturer they are using a genuine synthetic nicotine, otherwise a mixture would be assumed. A result of 100% would indicate the nicotine is tobacco-derived and the product has been misbranded (if labelled as SyN). From this regulatory perspective, we consider the method to be a definitive indicator with regard to the determining appropriate regulatory actions. The following text was added at the end of the "Radiocarbon Analysis" section to clarify this:

Line 358:

"As determined in this study, the radiocarbon results from nicotine analysis can fall under one of three scenarios:

1. pMC < 40 %: The test sample is confirmed to contain SyN.

2. pMC = 100 %: The test sample is confirmed to contain TDN.

3. pMC value falls between those in scenarios 1 and 2: The result is suggestive of the sample containing a mixture of both SyN and TDN and warrants further investigation.

From a regulatory standpoint, therefore, radiocarbon analysis of nicotine offers a definitive method for assessing the need for regulatory action with regards to nicotine products claiming to contain SyN."

The first line of the "Conclusions" was also modified to read (changes highlighted in red):

Line 413:

"Radiocarbon analysis offers a definitive method to differentiate tobacco-derived nicotine from synthetic nicotine for regulatory purposes."
